# Custom-made 3D PEEK plates versus 3D titanium plates for mandibular angle fracture fixation: a randomized clinical trial

**DOI:** 10.1186/s12903-025-07646-z

**Published:** 2026-01-30

**Authors:** Aliaa Osama Kamal, Ahmed Mamdouh Shaaban, Mohamed Mamdouh Shokry, Ibrahim Mohamed Abdelhamed

**Affiliations:** Department of Oral and Maxillofacial Surgery, Faculty of Dentistry, Alexandria University, Champlion Street, El-Azarita, Alexandria, Egypt

**Keywords:** Mandibular Fractures, Polyetheretherketone, Titanium, Bite Force

## Abstract

**Aim:**

This study aimed to determine the clinical and radiographic efficacy of custom-made 3D PEEK plates in comparison with 3D titanium plates for the management of mandibular angle fractures.

**Patients and methods:**

Eighteen patients with recent mandibular angle fractures indicated for open reduction and internal fixation were randomly allocated into two groups. The study group (*n* = 9) underwent fixation with custom-made 3D PEEK plates, while the control group (*n* = 9) received prebent 3D titanium plates adapted on virtually reduced models. Primary outcomes were bite-force recovery and bone-density (Hounsfield units, HU) at the fracture line measured on CT immediately post-op and at 12 weeks. Secondary outcomes included operative time, occlusion, interfragmentary stability and wound healing.

**Results:**

By the end of follow-up, Bite force increased significantly over time in both groups (*p* < 0.001) with no between-group differences at any time point. Mean bone density increased from 811.1 ± 68.2 HU to 1225.1 ± 111.7 HU in the PEEK group and from 809.0 ± 62.1 HU to 1248.3 ± 92.3 HU in the titanium group (*p* < 0.001); between-group differences were not significant. Operative time, occlusion outcomes and wound healing were similar between both groups.

**Conclusion:**

Custom-made 3D PEEK plates achieved comparable clinical, radiographic, and functional outcomes to 3D titanium plates, suggesting that PEEK is a viable alternative for mandibular angle fracture fixation.

**Trial registration:**

Trial registered at (ClinicalTrials.gov/NCT07156812/2025–08-28).

## Introduction

Maxillofacial injuries are among the most frequent conditions treated by oral and maxillofacial surgeons, with the mandible being the most commonly affected bone owing to its prominent anatomical position and functional role in mastication and occlusion [1]. Open reduction and internal fixation (ORIF) is the standard treatment for displaced mandibular fractures, but fixation at the mandibular angle remains challenging because of complex biomechanics and high torsional loads [2]. Three-dimensional (3D) miniplates were introduced to overcome limitations of conventional rigid and semirigid fixation. Their configuration provides multidirectional stability, resistance to torque, and favorable load distribution while maintaining a low profile and intraoperative adaptability [3].

Polyetheretherketone (PEEK) has emerged as an attractive alternative to titanium in craniomaxillofacial reconstruction due to several material-specific advantages. Its elastic modulus approximates that of cortical bone, potentially reducing stress shielding effects associated with titanium [4]. PEEK is radiolucent and does not produce CT or MRI artifacts, allowing more accurate assessment of fracture healing and surrounding tissues [5]. Additionally, PEEK is chemically inert, corrosion-resistant, and free of metal-related concerns such as cold sensitivity, metallic taste, and hypersensitivity reactions [6]. With the aid of computer-aided design and manufacturing (CAD/CAM), custom-made PEEK plates can be precisely fabricated to patient-specific anatomy, potentially enhancing fixation accuracy and reducing intraoperative adjustments [7, 8].

Although previous studies have evaluated titanium systems and experimental applications of PEEK in craniomaxillofacial surgery, evidence from randomized clinical trials comparing 3D patient-specific PEEK plates to conventional 3D titanium plates remains scarce. This study extends the current literature by directly comparing 3D customized PEEK fixation with commonly used 3D titanium miniplates.

This study aimed to assess the clinical, radiographic, and biomechanical outcomes of custom-made 3D PEEK plates for mandibular angle fracture fixation, with the hypothesis that they would provide comparable results to 3D titanium strut plates. Primary outcomes of the study include bite force recovery and bone density analysis of the fracture lines. The secondary outcomes assess intraoperative time, wound healing, and occlusion.

## Materials and methods

### Study design

**Fig. 1:**
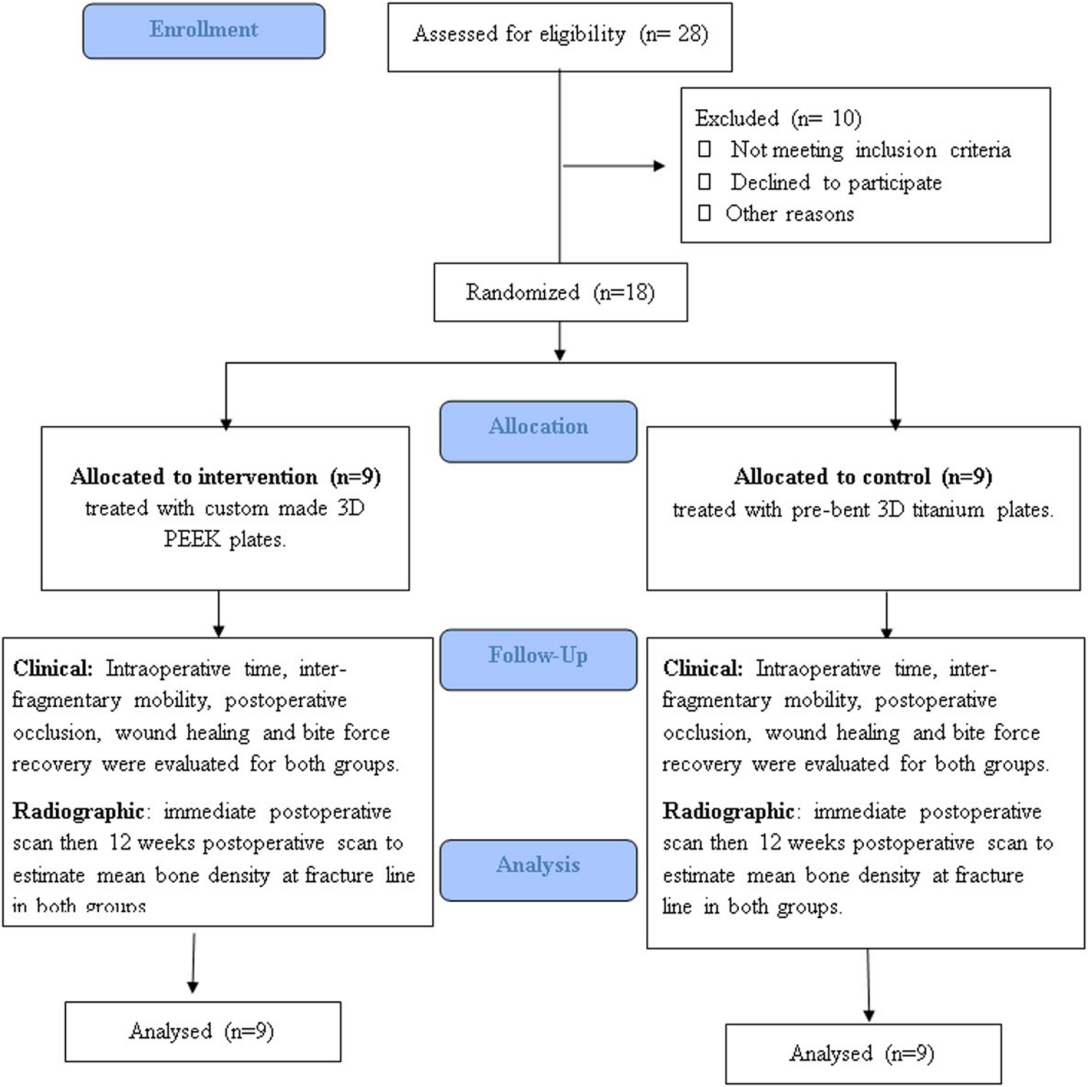
CONSORT flow chart

This study was a single-center, parallel-group randomized controlled trial conducted at the Oral and Maxillofacial Surgery Department, Alexandria University, between February 2022 to December 2023. The trial adhered to the CONSORT 2010 guidelines, and a flow diagram is provided (Fig. 1). The protocol was approved by the Ethics Committee of the Faculty of Dentistry, Alexandria University (IRB No. 00010556–12/2021), the study was designed prospectively, and registered retrospectively at(ClinicalTrials.gov/NCT07156812/2025–08–28).

Sample size was planned on 95% confidence level to detect differences in post-operative bone density between custom-made 3D PEEK and pre-bend titanium plates. Melek et al. [9]. reported mean ± SD bone density = 1658.15 ± 10.92 Hounsfield Unit (HU) when titanium plate was used, while Dessoky et al. [10] reported mean ± SD bone density = 900.40 ± 148.55 HU with PEEK plates. The calculated mean ± SD bone density difference = 757.75 ± 79.73 and 95% confidence interval = 658.79, 856.71. The required sample size was calculated to be 8 per group, increased to 9 to make up for cases lost to follow up. The total sample size = number of groups × number per group = 2 × 9 = 18 [11].

### Patients’ selection

The study included patients with recent mandibular angle fractures. Adult patients with fractures that require open reduction and internal fixation, without gender predilection who agreed to follow-up, and medically fit for general anesthesia were included in this study. Exclusion criteria included medically compromised patients, infected fracture lines, pathological or old fractures, completely edentulous patients, and comminuted fractures [12]. Patients provided informed consent outlining the procedures, its benefits, and challenges. They were allocated in a 1:1 ratio using an on-site computer software system with concealed allocation through sequentially numbered, opaque, sealed envelopes (SNOSE). Randomization was conducted with 2 & 4 random block sizes (http://www.randomizer.org/). Group A, 3D custom made PEEK plate were used and in Group B, pre-bent 3D titanium plates were used.

### Materials

**Fig. 2:**
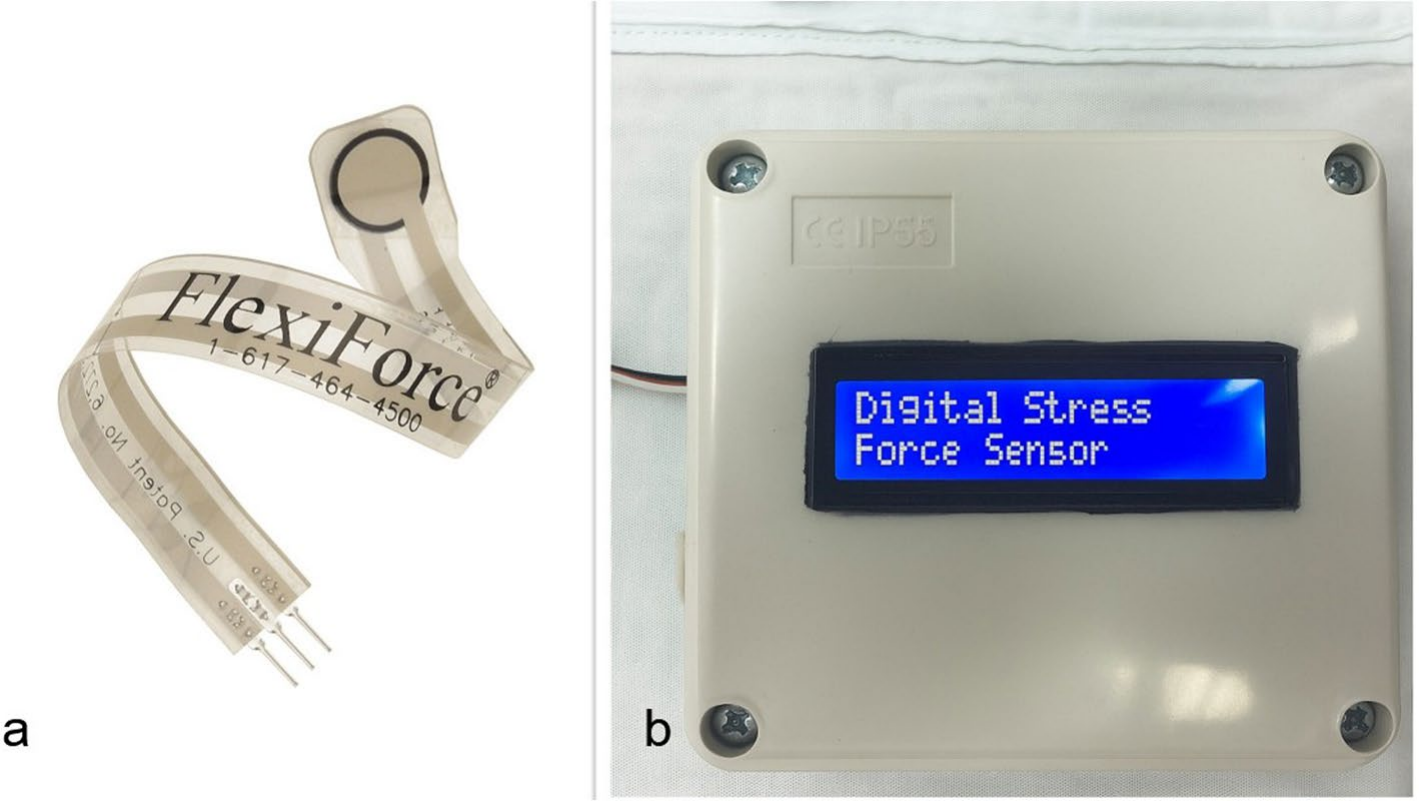
Piezoresistive force transducer sensor **(a**) Flexiforce A201 sensor, **b** circuit

1. Polyetheretherketone (PEEK)Disks (breCAM BioHPP,Bredent Medical GmbH & Co. KG, Senden, Germany.) Disk Diameter: Ø 98,5 mm. Disk thickness 16 mm.
2. Titanium plates and screws: 3-dimensional plates manufactured of pure titanium, 1.0 mm in thickness and secured with titanium screws. (Arab engineer’s co, Cairo, Egypt).
3. Piezoresistive force transduce sensor: FlexiForce A201 sensor (FlexiForce sensor, by Tekscan Boston, MA, USA.) (Fig. 2a) displaying a load range of 100 lb, equivalent to 440 N, and a sensitivity of 0.01 V/N. It is a flexible printed circuit planted within two polyester film layers reaching an ultimate thickness of 0.2 mm (Fig. 2b). The sensor’s active zone is 1 cm in diameter at its end, which is made of pressure-sensitive ink. The output readings from the transducer are in Volt (V), accordingly, the sensor was initially pre-habituated according to the manufacturer-recommended equation to make the expressed reading in Newton (N) [13].

### Preoperative phase

**Fig. 3:**
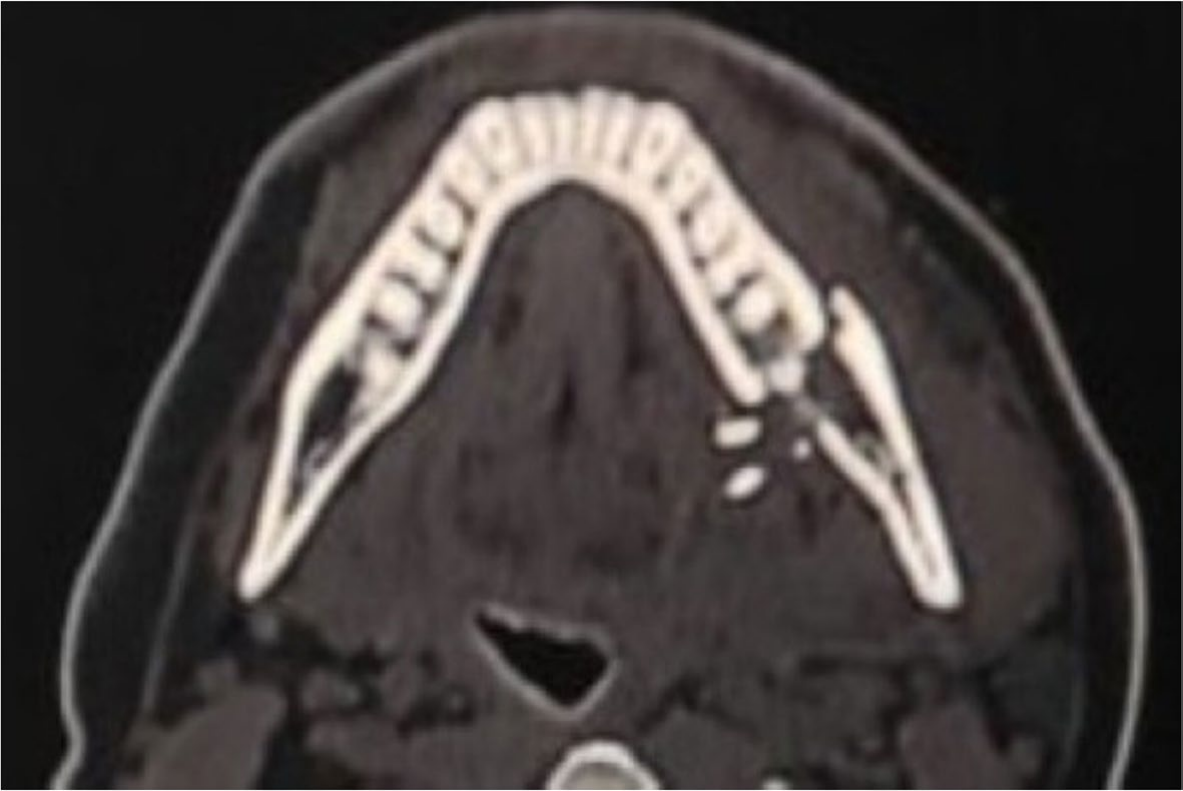
Preoperative axial view of CT-scan showing the fracture line

I. A comprehensive history taking and methodical clinical examination was implemented and logged for all of the enrolled cases. A Computed Tomography (CT) scan was conducted to determine the number and pattern of fracture lines, displacement severity, and the presence of teeth within the fracture line (Philips Brilliance 64 MDCT, Philips, Eindhoven, Netherlands) (Fig. 3).

II. Designing the PEEK Plate

Computer-guided virtual reduction was performed using specialized software (MIMICS; Materialise NV, Belgium) with surface-based rendering for surgical planning. CT images in DICOM format were imported into the software and segmented through thresholding to exclude soft tissues and highlight only bone and dentition. The mandibular bone and teeth were isolated, and a three-dimensional reconstruction was generated. The reconstructed mandible was segmented into distinct fracture fragments. which were then virtually reduced anatomically using the simulation module (repositioning option). The accuracy of reduction was verified in axial and coronal planes, and condylar position was confirmed relative to the glenoid fossa.

**Fig. 4:**
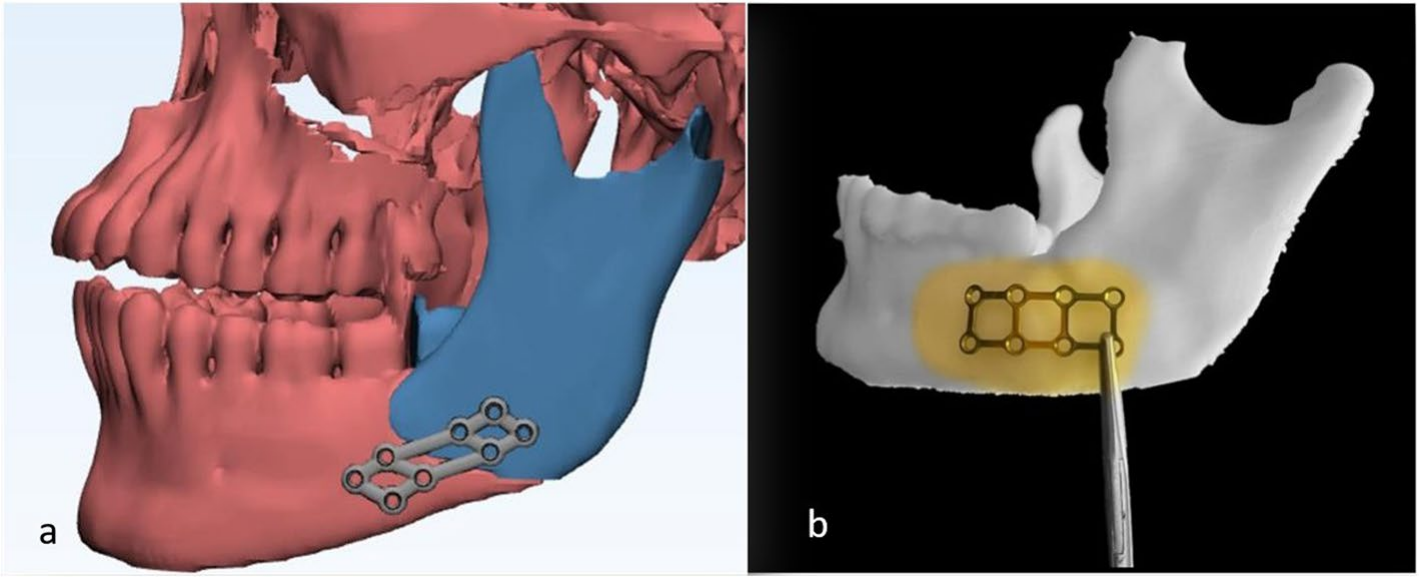
**a** Virtual reduction and design of the 3D PEEK plate, **b** Adaptation of the pre-bent 3D titanium plate

Following reduction, a 1-mm thick custom-made 3D plate was designed on the virtually reduced mandible (Fig. 4a). The design was exported in STL format to dedicated CAM software (INLAB CAM SW 16; Dentsply Sirona, USA) and milled using a five-axis milling machine (MC X5; Dentsply Sirona, USA) from a PEEK disk (bredent, breCAM.BioHPP, Germany). The fabricated plate was sterilized preoperatively. Screw hole distribution and strut configuration was planned to enhance load sharing across the mandibular angle while preventing excessive plate flexibility.

III. Construction of the virtually reduced model for pre-bending in the control group

For the control group, the virtually reduced mandible was printed as a stereolithographic model. A 1-mm thick 3D titanium strut plate was prebent on the model and sterilized for intraoperative use. Fixation used monocortical titanium screws (7-mm). (Fig. 4b).

### Surgical procedure

Patients underwent the required tests to obtain anesthesiologist clearance. All patients were treated under general anesthesia using nasotracheal intubation. The surgical site was prepared with povidone-iodine scrub solution, followed by draping with sterile towels to expose only the area of surgery. Access to the fracture was achieved through an extraoral submandibular incision approximately 4 cm in length, positioned 1.5–2 cm below the mandibular border. Standard layered dissection was performed through the platysma, deep cervical fascia, pterygomasseteric sling, and periosteum until the fracture site was adequately exposed. The bony segments were mobilized, any soft tissue interposed within the fracture line was cleared, and teeth located within the fracture were either preserved or extracted according to their condition. Temporary intermaxillary fixation (IMF) was applied to establish correct occlusion and to aid in reduction. Anatomical reduction was confirmed by direct visualization of proper alignment of the buccal cortex and inferior border. The surgical team was constant for all of the enrolled patients in this study, under supervision of the same main surgeon (AK). After exposure of the fracture lines one of the surgical team was entrusted with side selection and group distribution, by opening the sealed allocation envelope (IA).

**Fig. 5:**
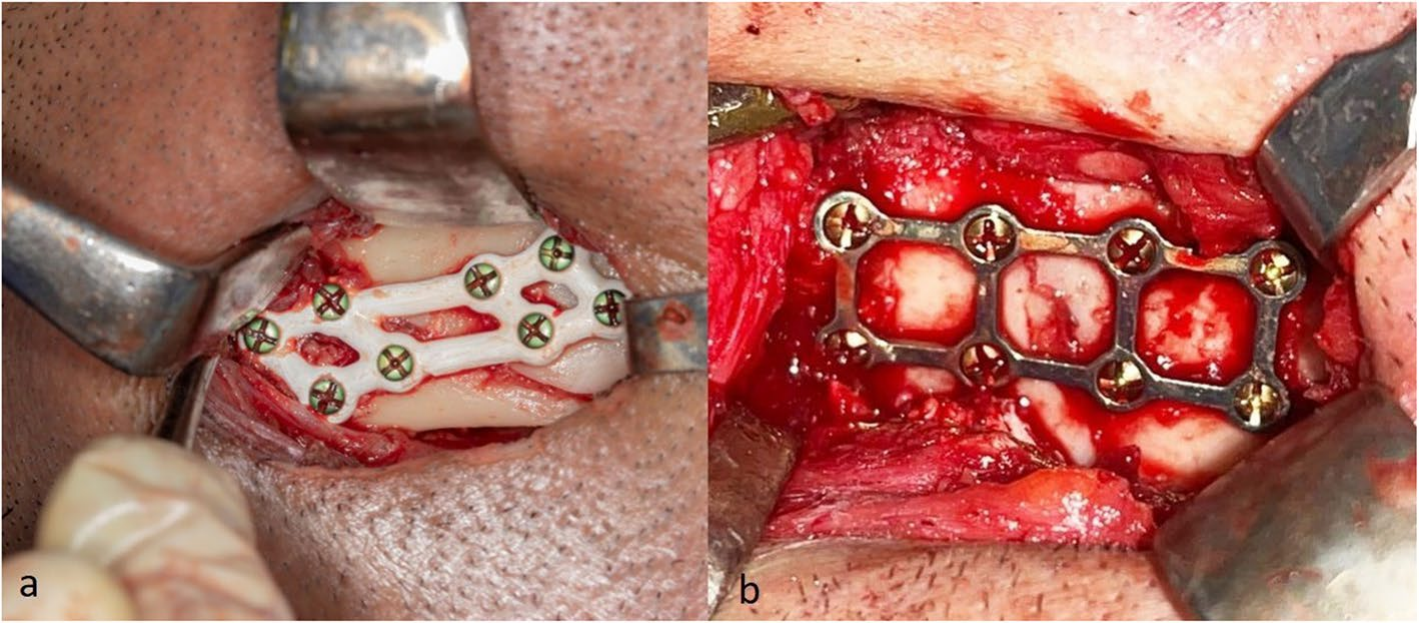
Clinical picture of fracture fixation (**a**) study group treated by 3D PEEK plate, **b** control group treated by 3D titanium plate

In study group (Group A), Custom-Made 3D PEEK plate was carefully positioned and secured with Mono-cortical Titanium screws (7 mm) across the fracture line to provide stabilization (Fig. 5a). For the control group (Group B). pre-bent 3D titanium plate and screws were used (Fig. 5b). The surgical wound was meticulously closed in layers, using Vicryl suture material (Johnson &Johnson Int. European Logistics Centre, Belgium). Patients were prescribed 875-mg amoxicillin + 125-mg clavulanic acid b.i.d for 5 days (Augmentin, GlaxoSmithKline, UK). Patients were instructed to apply an ice pack extra-orally for the first 24-h. Additionally, they were advised to adhere to a soft, high-protein, high-calorie diet for four weeks.

### Clinical variables

Clinical outcomes were evaluated through a rigorous follow-up schedule at 24-h, one, four, six and twelve postoperative weeks using several indices. Intraoperative time, Intra-fragmentary mobility (by bi-manual palpation across the fracture site), Postoperative occlusion (By checking the maximal intercuspal position (centric occlusion) to ensure proper occlusal relationship including molar relation, canine relation and midline centralization). Any occlusal disturbance including open bite or improper tooth contact was recorded. Wound healing is graded according to Southampton wound-grading system at 1, 7 and 14 days postoperatively [14].

Bite force was assessed by the same assessor (AK) using a piezoresistive force transducer sensor at the molar and premolar regions on the fractured side in both groups at 1, 6, and 12 weeks postoperatively. During each evaluation, patients were positioned upright with the head unsupported and directed forward in a natural posture. They were instructed to bite maximally on the sensor for a duration of 5 s. The 1-cm active zone of the sensor was sequentially placed in the first molar and first premolar regions on the ipsilateral side. To prevent direct contact with saliva, the sensor was enclosed in a disposable transparent plastic sheath, as it cannot withstand immersion or heat sterilization [15].

### Radiographic variables

**Fig. 6:**
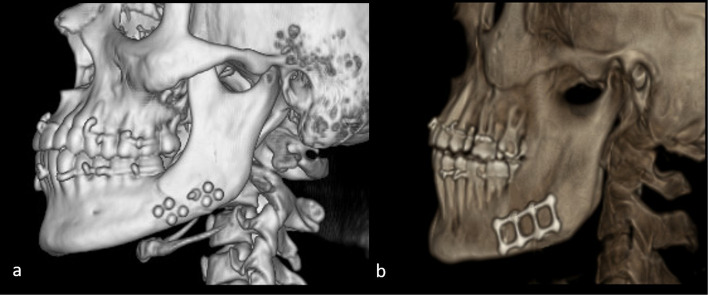
Three months postoperative 3D reconstruction view of CT-scan (**a**) 3D PEEK plate, **b** 3D Titanium plate

In both groups, a baseline CT scan was performed immediately postoperatively or on the following day to verify fracture reduction and fixation. A follow-up CT scan was obtained at 12 weeks to evaluate fracture healing and compare mean bone density with the immediate postoperative values (Fig. 6a,b). Bone density at the fracture site was assessed by selecting six points along the fracture line using CT software (3D Slicer image computing platform, 3D Slicer), and the mean of these measurements was calculated. All density values were expressed in Hounsfield Units (HU). Outcome assessors responsible for evaluating CT bone density measurements were blinded to group allocation to minimize assessment bias.

### Statistical analysis

Data were analyzed using IBM SPSS Statistics version 20.0 (Armonk, NY: IBM Corp). Normality was tested using the Shapiro–Wilk test. Quantitative variables were expressed as mean ± standard deviation, while qualitative variables were presented as frequencies and percentages. Comparisons between groups were made using Student’s *t*-test or Fisher’s exact test, as appropriate. Paired *t*-test and repeated measures ANOVA with Bonferroni correction were applied for within-group comparisons across time points. For non-normally distributed data, the Friedman test was used. A *p* value < 0.05 was considered statistically significant.

## Results

The study was conducted on 18 patients suffering from mandibular angle fracture, equally allocated into 2 groups. Demographic data are summarized in (Table 1). The mean intraoperative time was 2.29 h in the study group and 2.34 h in the control group, with no significant difference (*p* = 0.807). No interfragmentary mobility was reported in either group. All patients in the study group achieved satisfactory occlusion, while 2 patients in the control group (22.2%) developed mild occlusal derangement, which was corrected by selective grinding.

**Table 1:** Demographic data and epidemiology of the study (*n* = 18)

*n* = 18	**No. (%)**
Gender	
Male	15 (83.3%)
Female	3 (16.7%)
Age (years)	
Min. – Max	22.0–42.0
Mean ± SD	28.88 ± 7.57
EOT	
RTA	12 (66.7%)
IPV	4 (22.2%)
Falls	2 (11.1%)
Side	
Right	10 (55.6%)
Left	8(44.4%)
Other fracture	
No	13 (72.2%)
Zygomaticomaxillary Complex	2 (11.1%)
Zygomaticomaxillary Complex + Frontal	3 (16.7%)

*EOT* Etiology of Trauma, *RTA* Road Traffic Accident, *IPV* Inter Personal Violence

Wound healing was assessed using the Southampton wound-grading system on postoperative days 1, 7, and 14. In the study group, a statistically significant improvement was observed between day 1 and day 14 (*p* = 0.007). In contrast, the control group showed no statistically significant improvement over time (*p* = 0.054). When comparing between groups, however, there was no significant difference in healing scores at any time point.

Radiographic evaluation using immediate and 3-month postoperative CT scans confirmed satisfactory reduction and alignment of all fractures. Bone healing was assessed through bone density measurements in Hounsfield Units (HU). In the study group (PEEK plates), mean bone density increased significantly from 811.1 ± 68.24 HU immediately after surgery to 1225.1 ± 111.7 HU at 12 weeks (*p* < 0.001). Similarly, in the control group (titanium plates), bone density improved from 809.0 ± 62.1 HU to 1248.3 ± 92.27 HU (*p* < 0.001). No significant difference was found between the two groups at either the immediate postoperative scan (*p* = 0.947) or the 12-week follow-up (*p* = 0.638) (Table 2).

**Table 2:** Comparison between the two studied groups according to mean bone density across the fracture line

BONE DENSITY (HU)	Immediate postoperative	3 Months’ postoperative	P
Study (3D PEEK) (*N* = 9)			
Mean ± SD	811.1 ± 68.24	1225.1 ± 111.7	(< 0.001^*^)
Min. – Max	709.7–917.1	1052.2–1367.7	
Control (3D Titanium) (*N* = 9)			
Mean ± SD	809.0 ± 62.1	1248.3 ± 92.27	(< 0.001^*^)
Min. – Max	737.7–936.1	1076.3–1350.7	
P^1^	**0.947**	**0.638**	

*SD* Standard deviation

*p*: *p* value for comparing between immediate and 3 months postoperative

p^1^*p* value for comparing between the studied groups

^*^Statistically significant at *p* ≤ 0.05

Postoperative bite force was evaluated at 1, 6, and 12 weeks using a bite force gauge on the ipsilateral first molar and first premolar, with the mean value recorded in Newtons (N). Both groups showed a significant increase in bite force over time (*p* < 0.001 within groups). By 12 weeks, bite forces approached near-normal values. When comparing between groups, no statistically significant difference was found at any time point (week 1: *p* = 0.739, week 6: *p* = 0.571, week 12: *p* = 0.770) (Table 3).

**Table 3:** Comparison between the two studied groups according to bite force recovery across the three studied periods

Bite force recovery (N)	1week	6weeks	12weeks	P
Study (3D Peek) (*N* = 9)				
Min. – Max	89.0–132.0	181.0–233.0	281.0–324.0	< 0.001^*^
Mean ± SD	113.6 ± 14.22	202.7 ± 18.75	304.1 ± 14.50	
Control (3D Titanium) (*N* = 9)				
Min. – Max	93.0–136.0	180.0–238.0	277.0–345.0	< 0.001^*^
Mean ± SD	111.3 ± 13.57	207.7 ± 17.93	306.7 ± 21.26	
P^1^	**0.739**	**0.571**	**0.770**	

*SD* Standard deviation

p *p* value for comparing between the three studied periods

p^1^ *p* value for comparing between the studied groups

^*^Statistically significant at *p* ≤ 0.05

## Discussion

With the advances in virtual surgical planning, patient-specific fixation devices have gained increasing popularity due to their superior adaptability and predictable outcomes. Among the available materials, poly-ether-ether-ketone (PEEK) has attracted attention because of its strength, biocompatibility, radiolucency, and biomechanical resemblance to cortical bone [16]. The present study compared the performance of custom-made 3D PEEK plates with pre-bent 3D titanium plates in the fixation of mandibular angle fractures, evaluating clinical, radiographic, and biomechanical outcomes.

The demographic analysis showed a mean age of 28.88 ± 7.57 years. These findings are consistent with Mabrouk et al. [17], who reported a mean age of 25.7 years among Egyptian patients with maxillofacial fractures. This reflects the higher risk in the third and fourth decades, when activity levels and exposure to trauma are greatest. 83.3% of the patients were male, in line with Kamel et al. [18], who found a 12.6:1 male-to-female ratio. The male predominance is explained by greater involvement in high-risk jobs, disputes, and aggressive behaviors. Road traffic accidents (RTAs) were the main cause of mandibular fractures, accounting for 66.7% of cases. Reckless driving, poor compliance with safety rules, and weak enforcement of traffic laws likely explain this high incidence [19].

In both groups, mandibular angle fractures were exposed using an extraoral submandibular approach, and all incisions healed uneventfully without dehiscence, consistent with previous reports [20]. This approach provides superior access, facilitates accurate reduction, and allows meticulous debridement before fixation. It has also been associated with easier application of 3D plates and lower complication rates, including reduced wound dehiscence, infection, pain, and edema, when compared with intraoral techniques [21].

Intraoperative time was recorded for all cases, and no significant difference was detected between the two groups (*p* = 0.807). Earlier reports have shown that the use of custom-made PEEK plates can help shorten operative time, while pre-bending titanium plates has also been noted to achieve a similar effect [5, 22]. Shorter surgeries are beneficial as they are associated with fewer postoperative complications such as pain, swelling, and infection, and they also help reduce operating room expenses, which remain a major component of trauma care costs.

None of the patients demonstrated interfragmentary mobility postoperatively, in agreement with Xue et al. [23] who found that 3D plates provide superior stability. Biomechanical studies have explained this by showing that the 3D strut plate design distributes stresses more effectively than linear arrangements, allowing the plate to better resist torsional forces and thus enhance stability [24]. Patient-specific PEEK plates in linear designs have also been reported to maintain adequate stability and eliminate mobility in mandibular fractures [25]. The 3D configuration of the PEEK plates in the present study may have further reinforced this outcome.

With respect to postoperative occlusion, all patients in the study group achieved satisfactory intercuspation, whereas two patients in the control group (22.2%) developed mild occlusal discrepancies. This difference was not statistically significant and agrees with the findings of Abaas et al. [5]. Who reported no significant difference between PEEK and titanium plates in mandibular body fracture fixation. The rate of occlusal derangement observed here is also comparable with previous reports on 3D plates, which ranged from 0–20% [26]. This favorable outcome is attributed to the enhanced stability provided by the 3D plate design, which maintains accurate alignment of the fracture segments and thus promotes stable occlusion.

Overall, extraoral wound healing was assessed using the Southampton system (Bailey & Love, 25th edition) [27]. When comparing wound healing scores across time points within each group, a statistically significant improvement was observed in the study group between day 1 and day14 (*p* = 0.007). In contrast, the control group did not show a significant change (*p* = 0.054), likely due to one patient who developed a serous discharge at two weeks, which delayed complete healing and influenced the scores. This highlights a limitation of the Southampton wound grading system, as even minor complications such as localized discharge can markedly affect results. No significant difference was observed between the two groups at any of the evaluated time points. Wound healing depends on multiple factors beyond the choice of fixation hardware, including: aseptic technique, incision design, fracture stability, suturing method, comorbidities, and lifestyle habits. In the present study, strict infection control, standardized incision type, unbiased randomization, and careful tension-free suturing likely contributed to the comparable healing outcomes between groups.

CT scans allow accurate assessment of bone mineral density, expressed in Hounsfield units, and have been widely used to monitor fracture healing [28]. In this study, preoperative, immediate postoperative, and 12-week postoperative CT scans were obtained. Bone density was measured by taking six readings around the fracture line on each scan, and their averages were calculated to determine the mean values.

At the end of follow-up, both groups showed a significant increase in bone density from the immediate postoperative to the 12-week scan (p < 0.001). These findings are consistent with Dessouky et al. [10] and Abdelmoneim et al. [25] who reported significant postoperative increases in bone density when using PEEK plates in mandibular fractures. The ability of PEEK to maintain sufficient stiffness and stability may explain these outcomes. Our results also align with Chrcanovic and Ramos [29]. Who noted that 3D plates generally achieve superior stability and demonstrated significant bone density improvement in angle fractures. No statistically significant difference in mean bone density was observed between the two groups in the 12th week postoperative scan (*p* = 0.638), these results suggest that both custom-made 3D PEEK plates and pre-bent 3D titanium plates provide adequate stabilization to support satisfactory bone healing and comparable postoperative bone densities.

Bite force is generated by the action of the jaw elevator muscles under central nervous system regulation, influenced by mechanoreceptors, nociceptors, craniomandibular biomechanics, and reflex mechanisms. For this reason, bite force measurement is a useful tool in maxillofacial surgery to assess the functional outcomes of different osteosynthesis techniques [30]. In the present study, a piezoresistive force transducer sensor was used to record bite force [13]. Within each group, bite force values at the fracture site showed a significant increase from 1 to 6 and 12 weeks postoperatively (*p* < 0.001).These findings agree with Lovald et al. [31]demonstrated that 3D plates are biomechanically capable of supporting masticatory forces during healing. Saxena et al. [32] also reported significant improvement in bite force overtime with 3D fixation systems. No statistically significant differences in bite force values were observed between the two groups at any time point. This shows the favorable mechanical behavior of Custom -made 3D PEEK plates, which provide superior stability and effective force distribution similar to 3D titanium plates. It is important to note that several factors can affect postoperative bite force values, including age, dental status, and the presence of malocclusion. For instance, patients with periodontal involvement often exhibit impaired sensory feedback, leading to reduced bite force [33].

The results of this study reinforce the potential of custom-made 3D PEEK plates as a viable alternative to titanium fixation systems in mandibular angle fractures as both fixation systems revealed comparable bone healing outcomes. Although PEEK possesses lower stiffness than titanium, optimizing screw distribution and strut configuration likely compensated for this difference, resulting in comparable load resistance during functional recovery [5]. Additionally, PEEK’s modulus of elasticity, which is closer to that of cortical bone, may enhance load sharing across the fracture site and minimize stress shielding, thereby supporting physiological bone healing [4]. The use of CAD/CAM workflows for patient-specific PEEK plate fabrication enables highly accurate anatomical conformity, minimizing the need for intraoperative modification and supporting greater precision during fixation [34].

Although fixation stability and bone healing outcomes were statistically equivalent between the two materials, the findings are clinically meaningful. Demonstrating comparable performance indicates that patient-specific PEEK plates can achieve fixation outcomes similar to titanium while offering unique advantages such as radiolucency, absence of imaging artifact, and avoidance of metal-related sensitivities. PEEK also offers favorable biocompatibility, chemical stability, and resistance to sterilization methods including autoclaving and plasma sterilization. These attributes may enhance postoperative assessment, accurate radiographic evaluation, and patient comfort [6, 35].

It is known that the outstanding advantage of PEEK lies in its compatibility with additive manufacturing [36], the plates in this study were milled from pre-fabricated PEEK disks due to local manufacturing constraints. Milling increases production steps and does not fully exploit the precision or efficiency of 3D printing. Future investigations should evaluate fully 3D-printed PEEK plates, which may further enhance customization, reduce material waste, and streamline production. Additionally, the higher material and fabrication costs remain a consideration for general clinical adoption.

This study has some limitations. Firstly, the intervention compared patient-specific 3D PEEK plates with pre-bent 3D titanium plates rather than fully custom 3D-printed titanium plates, which limits the extent to which differences can be attributed specifically to patient-specific design. Additionally, the sample size was calculated to detect differences in bone density and was not intended to evaluate infrequent clinical complications; therefore, the study is underpowered for safety outcomes and any observations regarding complications should be interpreted with caution. Finally, although the 12-week follow-up period is appropriate for assessing early healing and fixation stability, it does not capture longer-term outcomes such as hardware fatigue, late complications, or bone remodeling. In addition, the small sample size and short-term assessment window limit the generalizability of the findings. Further studies with larger cohorts and extended follow-up are required to confirm the long-term effectiveness and stability of PEEK fixation systems.

## Conclusion

Custom-made 3D PEEK plates provide clinical and radiographic outcomes equivalent to 3D titanium plates in mandibular angle fracture fixation. Their patient-specific design and radiolucency offer potential advantages, though higher cost and limited availability remain barriers to widespread use.

## Data Availability

Datasets are available from the corresponding author on reasonable request.

## Abbreviations

IMF: Inter-maxillary fixation
ORIF: Open reduction and internal fixation
*CT*: Computed Tomography.
*SNOSE*: Sequentially Numbered, Opaque, Sealed Envelopes.
*HU*: Hounsfield Units.
*PEEK*: Polyetheretherketone
*3D*: Three-Dimensional
